# Deep Neural Networks for Behavioral Credit Rating

**DOI:** 10.3390/e23010027

**Published:** 2020-12-27

**Authors:** Andro Merćep, Lovre Mrčela, Matija Birov, Zvonko Kostanjčar

**Affiliations:** 1 Laboratory for Financial and Risk Analytics, Faculty of Electrical Engineering and Computing, University of Zagreb, 10000 Zagreb, Croatia; lovre.mrcela@fer.hr (L.M.); zvonko.kostanjcar@fer.hr (Z.K.); 2 Privredna Banka Zagreb, Member of Intesa Sanpaolo Group, 10000 Zagreb, Croatia; matija.birov@pbz.hr

**Keywords:** deep neural network, credit rating, credit risk assessment, behavioral model

## Abstract

Logistic regression is the industry standard in credit risk modeling. Regulatory requirements for model explainability have halted the implementation of more advanced, non-linear machine learning algorithms, even though more accurate predictions would benefit consumers and banks alike. Deep neural networks are certainly some of the most prominent non-linear algorithms. In this paper, we propose a deep neural network model for behavioral credit rating. Behavioral models are used to assess the future performance of a bank’s existing portfolio in order to meet the capital requirements introduced by the Basel regulatory framework, which are designed to increase the banks’ ability to absorb large financial shocks. The proposed deep neural network was trained on two different datasets: the first one contains information on loans between 2009 and 2013 (during the financial crisis) and the second one from 2014 to 2018 (after the financial crisis); combined, they include more than 1.5 million examples. The proposed network outperformed multiple benchmarks and was evenly matched with the XGBoost model. Long-term credit rating performance is also presented, as well as a detailed analysis of the reprogrammed facilities’ impact on model performance.

## 1. Introduction

Losses caused by borrowers defaulting on their credit obligations are part of the banks’ normal operating environment. The number and severity of default events can vary over time, which affects the variance of experienced losses. Unanticipated shocks can have especially devastating consequences; for example, the global financial crisis caused massive losses to the financial sector. The Basel regulatory framework, now in its third installment, was introduced to improve banks’ ability to absorb such shocks by defining various capital, transparency, and liquidity requirements [1].

**Figure 1:**
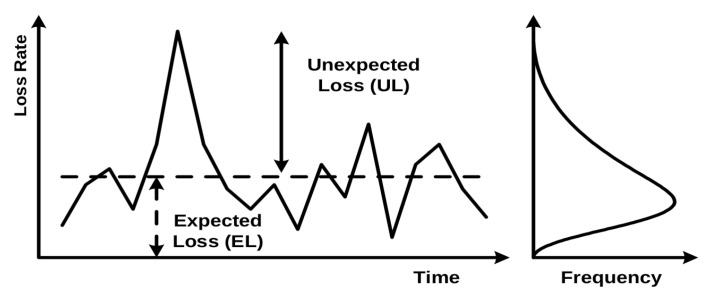
Illustration of variance in experienced losses (left) and distribution of losses (right) [2].

Figure 1 illustrates banks’ experienced losses over time. Future annual losses of a bank are, of course, impossible to know, but it is possible to estimate the average level of credit losses using long-term historical data. These are called Expected Losses (ELs) and are illustrated by the dashed line in Figure 1; values above the line are called Unexpected Losses (ULs) [2]. ELs are typically covered from annual revenues (i.e., managed through the pricing of credit exposures and provisioning), while ULs are charged against the capital (since it is unlikely that a bank will be able to completely cover them by revenue alone) [1].

It is possible to estimate expected loss using individual components of a portfolio. There are three key factors:

- Probability of Default (PD): the average percentage of the defaulted obligors for a rating grade,
- Loss Given Default (LGD): share of the exposure the bank might lose in case of a default, and
- Exposure At Default (EAD): estimated outstanding amount in case of a default, i.e., total value to which the bank is exposed.

LGD is a percentage of EAD and is primarily dependent on the type and amount of collateral [2]. EL can be expressed as:

(1) EL=PD·LGD·EAD.

The Internal Ratings-Based approach (IRB) [3] adopted for the Basel framework, defines two methodologies: foundation and advanced. All banks are required to provide their supervisors with an internal estimate of the PD regardless of the methodology used. The difference is that banks estimate LGD and EAD only in the advanced methodology, while the foundation methodology provides their estimates through the use of standard supervisory rules [3]. Although there is some research on LGD and EAD (such as [4,5,6,7]), the primary focus of this paper will be the significantly more popular topic of PD estimation.

From a model development perspective, we can identify two basic kinds of models depending on their purpose: application and behavioral. Application models are used during the loan application phase, meaning that their task is to approve acceptable clients and reject those that are likely to default in the future. The purpose of behavioral models, on the other hand, is to assess the future performance of an existing credit portfolio of a bank. Logistic regression represents the industry standard for both, and the type of training data defines whether it will be used as an application or a behavioral model.

Application model development is the more common setting in existing research, and it is usually carried out using publicly available datasets. Most of the published papers in the field use one or more of the publicly available application datasets, such as the UCI credit datasets [8] or Kaggle Give Me Some Credit competition data [9]. To put things into perspective, out of 187 papers in the review paper [10], forty-five percent used Australian or German UCI credit datasets. There are exceptions, however, as some researchers develop corporate credit rating models (instead of focusing on more common retail clients), and others collaborate with financial institutions and gain access to proprietary data. The size of the development sample also varies significantly, ranging from less than 1000 examples (e.g., UCI datasets, corporate data), up to 150,000 in the case of Give Me Some Credit data (with a couple of proprietary datasets containing more examples).

While commercial credit rating models are, as we have previously mentioned, usually based on logistic regression, other machine learning algorithms were applied for this class of problems. Support vector machines were applied to UCI German and Australian datasets as a standalone model in [11], with a modification as a weighted least squares SVM. The combined SVM and random forest classifier was tested on the same two UCI datasets in [12]. Reference [13] proposed a fuzzy SVM approach that outperformed several other models on a corporate dataset of 100 companies.

Ensemble models, both boosting and bagging based, were employed as well. Reference [14] proposed a boosted CART ensemble for credit scoring. The gradient boosting model outperformed several other benchmarks on a dataset of 117,019 examples in [15]. Taiwanese financial institution provided a dataset (6271 examples, 9.58% default rate) for the development of an XGBoost model in [16]. Several ensemble models were developed for the UCI credit card clients dataset in [17]. A comprehensive overview of classification algorithms trained on publicly available data is available in [18]. The authors measured the performance of a large number of models, ranging from simple individual classifiers to complex ensembles, and based on their research results, recommended random forest as a benchmark for future model development.

Deep neural networks combined with clustering algorithms were applied to the Give Me Some Credit dataset in [19]. Credit card delinquencies were predicted using a neural network in [20]; the fairly large dataset (711,397 examples, 0.92% delinquency rate) was provided by a bank in Brazil. Another deep model achieved the highest AUC score on Survey of Consumer Finances (SCF) data [21]. The deep belief network model in [22] outperformed multiple benchmark models on the CDS contract data of 661 publicly-traded firms. The feedforward network was trained on a corporate sample of 7113 Italian small enterprises in [23]. More recently, a self-organizing neural network model demonstrated superior performance on a large French corporate bankruptcy dataset in [24].

The assessment of an existing portfolio using a neural network was presented in [25]. The proposed network was used to model a transition function of a loan from one state to the other. It was trained on a very large dataset of 120 million mortgage records originating across the U.S. between 1995 and 2014, with features that describe each loan and its month-to-month performance.

Recent work includes applications of machine learning models on alternative sources of data. Reference [26] proposed an LSTM model for peer-to-peer lending. The model was trained on 100,000 examples with features describing online operation behavior and other credit data. Convolutional neural networks were used for mortgage default prediction in [27]. The dataset of 20,989 examples was provided by Norway’s largest financial services group DNB, with features that included daily balances of clients’ checking accounts, savings accounts, credit cards, and transactional data.

While it is apparent that a large number of different non-linear models have been studied in the field of credit risk assessment, regulatory requirements for the explainability of the model output are among the main reasons why logistic regression still represents the industry standard. In our view, removing this limitation and allowing the development of more complex and accurate models would be beneficial to both banks and consumers. In the case of application models, the increased accuracy of the rejection of bad loans and the approval of good ones is in the interest of both banks and their potential clients. Accurate behavioral models could be used as an early warning mechanism, which could allow banks to issue loans with better terms to clients who are likely to default in the near future. We believe that thorough research on larger datasets could provide a better understanding of complex models than those developed on smaller, publicly available samples. Additionally, most of the existing research deals with application models, while behavioral models are still largely uncharted territory. To that extent, we develop a deep learning model for behavioral credit risk assessment as we believe that it has the potential to capture the complex dependencies between input features and target labels on a large amount of data. The behavioral development sample we use includes more than 1.6 million examples spanning ten years, from 2009 to 2018. These data allow us to measure model performance during and after the global financial crisis, as well as long-term model performance. Insights from [18] were taken into account when writing this paper—both recommended benchmark models and the most useful performance measures.

The following section of the paper offers the dataset description. The models and methods are covered in Section 3, and Section 4 offers a list of all relevant performance measures. Finally, the results are presented in Section 5.

## 2. The Data

This research is a collaboration with a large Croatian bank that provided a proprietary model development sample, which was used in this paper. The sample is a behavioral credit risk dataset that represents a part of Banks’ portfolio between 2009 and 2018. Each example in the dataset is a snapshot of the information on a loan (also called facility) created at the end of each year (i.e., 31 December is the snapshot date for all years from 2009 to 2018). Note that the data contain different examples that describe the same facility on different snapshot dates. Every snapshot contains features from the following categories:

- Tenure features, which contain data on the length and volume of the business relationship of the client and the bank,
- Data on the balance of current and business accounts, the balance of deposit, and regular income,
- Features that measure the average monthly obligations of the client, as well as the average monthly burden (debt burden ratio),
- The client’s utilization of an overdraft,
- Features that describe the credit history of the client (days past due and debt),
- The balance of the current account.

All these features are monitored during the observation period of one year that precedes the snapshot date. We trained all models to predict events of default within the performance period, which is defined as 12 months after the snapshot date.

A facility is considered to be in default if it is past due more than 90 days on credit obligation (this definition is in accordance with Basel III [28]). In our case, it follows that the defaulted contracts are the ones that are more than 90 days past due during the performance period. We labeled the defaulted facilities as the positive class (or one), while non-defaulted ones were given the negative label (or zero); in machine learning terms, this is a formulation of a binary classification problem. Note that this defines a default on a facility level instead of the client level; clients with multiple loans may have some loans in default and other loans labeled as non-defaults.

Reprogrammed facilities are loans whose terms and conditions have changed in order to ease the debt repayment process. The original loan (reprogram) is closed, and a new loan with better terms is issued. The new loan has the negative label, while the reprogrammed loan can either be labeled as default or non-default; both cases will be examined in this paper.

Some facilities were excluded from the model development sample. All examples that were in default on the snapshot date were removed, as well as all facilities that were in default status at any time nine months prior to the snapshot date.

The dataset implicitly assumes that all examples are independent, meaning that data for the same facility on two different snapshot dates represent two different training examples. This results in a common setting in binary classification problems: the input is represented with a vector, and the output is a single scalar value (either zero or one).

**Figure 2:**
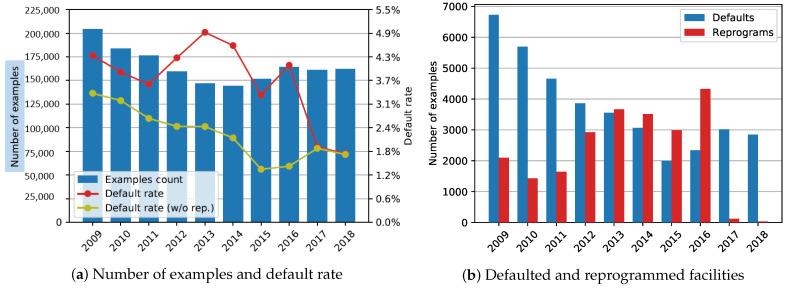
End-of-year dataset statistics: (**a**) the number of examples and the default rate (with reprogrammed facilities labeled as both defaults and non-defaults); (**b**) number of defaulted and reprogrammed loans for each snapshot date.

The exact number of facilities and the default rate (with and without reprograms) at the end of each year are shown in Figure 2a. The number of defaults and reprograms compared on a yearly basis is shown in Figure 2b.

We decided to split the available data into two separate datasets. The first one includes facilities from 2009 to 2013 and the second dataset those from 2014 to 2018. This allowed us to compare models trained on the data during the global financial crisis with ones trained on more recent data. We also are able to measure the long-term performance of models from the first group.

The last year of each dataset was isolated as a time-disjoint dataset, which was used for measuring model performance; we call it the out-of-time dataset. Remaining years (in-time dataset) are used for model development and validation.

In the case of the 2009–2013 dataset, we have 870,710 in total with a default rate of 4.16%. The in-time dataset contains 723,825 examples with a default rate of 4.01%, while the out-of-time portion has 146,885 with a significantly higher default rate of 4.91%.

The 2014–2018 dataset contains 782,875 facility snapshots with a default rate of 3.10%. The in-time subset has a higher default rate at 3.44% (and 620,646 examples). The out-of-time dataset in 2018 has the lowest default rate of only 1.77% and 162,229 examples.

We used in-time data to determine which features will be used. All features that had more than 50% missing values were removed, as well as those that contain the same value in more than 80% of examples. This resulted in 109 and 108 features for the 2009–2013 and 2014–2018 datasets, respectively.

## 3. Models and Methods

This section contains a brief description of all models used within the paper. For more details, the reader can refer to the included references.

### 3.1. Logistic Regression

Logistic regression, also known as the logit model, is the industry standard for credit risk modeling. It is used for the analysis of binary dependent variables or, in machine learning terminology, solving binary classification problems. Given an example $x=(x_{0},x_{1},\ldots ,x_{n})$ from the input space $R^{n}$, logistic regression models the posterior probability of the positive class $C_{1}$ as:

(2) $$p\left(C_{1}|x\right)=h\left(x|\beta_{0},\beta \right)=\sigma \left(\beta_{0}+\beta^{T}x\right),$$

where σ(z) is the sigmoid or logistic function:

(3) $$\sigma \left(z\right)=\frac{1}{1+e^{-z}}.$$

If we introduce a fixed dummy feature $x_{0}=1$ for each input example, (2) can be simplified to $p\left(C_{1}|x\right)=\sigma \left(\beta^{T}x\right)$, where $\beta =(\beta_{0},\beta_{1},\ldots ,\beta_{n})$. For a dataset $D={\left\{x^{\left(i\right)},y^{\left(i\right)}\right\}}_{i=1}^{N}$ with binary labels $y^{\left(i\right)}$, the likelihood function can be expressed as:

(4) $$L\left(\beta \right)=\prod_{i=1}^{N}{\left[h(x^{\left(i\right)}|\beta )\right]}^{y^{\left(i\right)}}\cdot {\left[1-h(x^{\left(i\right)}|\beta )\right]}^{1-y^{\left(i\right)}}.$$

Applying the negative logarithm to the likelihood function gives us the binary cross-entropy error:

(5) $$-\log L\left(\beta \right)=-\sum_{i=1}^{N}\left\{y^{\left(i\right)}\cdot \log\sigma \left(\beta^{T}x^{\left(i\right)}\right)+\left(1-y^{\left(i\right)}\right)\log\left(1-\sigma \left(\beta^{T}x^{\left(i\right)}\right)\right)\right\}.$$

Note that the same error function will be used later for the neural network models. Parameters β that minimize the cross-entropy error function are usually obtained by gradient methods, e.g., gradient descent [29].

### 3.2. Support Vector Machine

Just like logistic regression, the support vector machine model is also a well-known linear model, although it works in a slightly different manner. Consider a binary classification problem with a dataset that is linearly separable in the feature space, with modified class labels $y\in \left\{-1,+1\right\}$ (we previously mentioned 0 as a negative class label). It is likely that there are multiple solutions in terms of the typical linear model $h\left(x\right)=w^{T}x+b$, all of which separate the two classes perfectly. The Support Vector Machine (SVM) model chooses the decision boundary that has the largest possible separation, the so-called margin, between classes. Since the decision boundary is defined by h(x)=0, a model that separates classes perfectly will satisfy the constraints $h(x^{\left(i\right)})\geq 0$ if $y^{\left(i\right)}=+1$ and $h(x^{\left(i\right)})<0$ if $y^{\left(i\right)}=-1$. These can be rewritten as $y^{\left(i\right)}\cdot h\left(x^{\left(i\right)}\right)\geq 0$ for all i=1,2,…,N. The distance from the decision boundary is equal to:

(6) $$d^{\left(i\right)}=\frac{\left|h(x^{\left(i\right)})\right|}{∥w∥}=\frac{y^{\left(i\right)}\cdot \left(w^{T}x^{\left(i\right)}+b\right)}{∥w∥}.$$

Note that scaling the parameters w and *b* does not change the distance (6), allowing scaling such that $y^{\left(i\right)}\cdot \left(w^{T}x^{\left(i\right)}+b\right)=1$ holds for the point that is closest to the surface [29]. Since the margin is defined as the distance to the closest example, using (6), it follows that the margin is equal to 1/∥w∥ after the aforementioned scaling. Maximizing 1/∥w∥ is equivalent to minimizing ${∥w∥}^{2}$, so the optimization problem can be written as:

(7) $$\begin{array}{cc} \; & \underset{w,b}{arg min}\;\frac{1}{2}{∥w∥}^{2} \end{array}$$

(8) $$\begin{array}{cc} \mathrm{subject}\;\mathrm{to}\; & y^{\left(i\right)}\cdot \left(w^{T}x^{\left(i\right)}+b\right)\geq 1,\;\forall i. \end{array}$$

The solution for the optimization problem can be obtained by constructing the Lagrangian function. Eliminating parameters w and *b* from the Lagrangian gives the dual representation of the margin maximization problem and introduces the kernel function. It is worth mentioning that combining SVM with an appropriate kernel function, such as the Gaussian radial basis, creates a powerful non-linear classifier. For more details, refer to [29,30].

### 3.3. Random Forest

We chose two different ensemble models as non-linear benchmarks: random forest (a recommended benchmark model in [18]) and a gradient boosting-based model, which is presented in Section 3.4.

To understand the random forest model, we have to introduce several concepts first, starting with CART [31]. Classification And Regression Trees (CART) partition the feature space into two regions and model the predictions as either the average or majority vote of samples in each region. The feature space can be split further by adding depth to the trees; this process lasts until an appropriate stopping criterion is met. The complexity of the CART model can vary with tree depth; trees with many levels will probably overfit the data, and shallow trees will likely exhibit high bias.

High-variance models, such as deep CARTs, are well suited for the bagging technique [32]. Bagging or bootstrap aggregating trains the same model (or tree if we use CART) on bootstrapped samples of the available training data. The overall model result is then a simple average for regression, or a majority vote for classification tasks. This averaging of approximately unbiased models reduces their high variance, which is expected to improve the ensemble’s generalization performance. If we assume that the outputs of *B* trees, each with variance $\sigma^{2}$, are identically distributed with positive pairwise correlation ρ, the variance of the average is [30]:

(9) $$\rho \sigma^{2}+\frac{1-\rho }{B}\sigma^{2}.$$

Increasing the number of trees *B* reduces the second term only, meaning that further reduction of variance requires decorrelating trees. The random forest algorithm [33] reduces the correlation by selecting a random subset of *m* features as candidates for each new node split when growing trees on the bootstrapped dataset. The number of candidates *m* should be less than or equal to the total number of features *n*; a commonly used value is $m=\sqrt{n}$. For more information, refer to [30,33].

### 3.4. Gradient Boosting

Unlike random forests, which use high variance models as base predictors and reduce ensemble variance through averaging of individual outputs, boosting aims to create a powerful model from weak base learners in an additive manner.

In the supervised learning setting, with training set ${\left\{\left(x^{\left(i\right)},y^{\left(i\right)}\right)\right\}}_{i=1}^{N}$, we generally seek to minimize some loss function L(y,h(x)). In the case of gradient boosting, the hypothesis function *h* is defined as a weighted sum of outputs of *B* weak base learners $b_{i}\left(x\right)$ (all of which belong to a class B, such as shallow CART):

(10) $$h\left(x\right)=\sum_{k=1}^{B}\beta_{k}b_{k}\left(x\right).$$

The pseudocode for training a gradient boosting ensemble from [34] is available in Algorithm 1. We will use the Python package XGBoost (Documentation available at https://xgboost.readthedocs.io/) for training the benchmark model.
**Algorithm 1:** Gradient boosting [34].
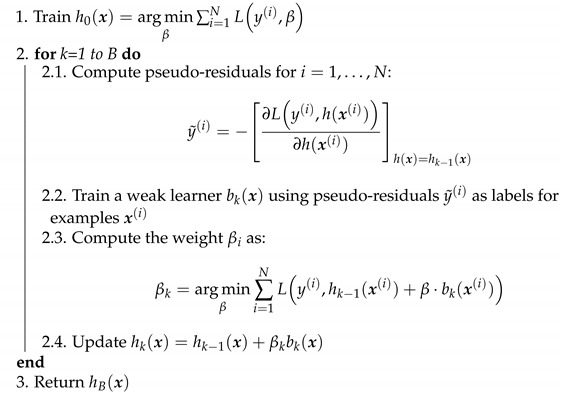


### 3.5. Feedforward Neural Network

The feedforward neural network is the oldest artificial neural network architecture; some models such as multilayer perceptron were defined as early as 1962 [35]. “Feedforward” refers to the lack of recurrent connections between neurons, i.e., information moves only forward through the network. It uses neurons that receive their input x via *n* input features $x_{1},\ldots ,x_{n}$, with an addition of a fixed feature $x_{0}=1$ (also known as the dummy feature). A neuron’s output *f* is computed by applying a non-linear activation function ϕ to a linear combination of input features with their corresponding parameters: weights $w_{1},\ldots ,w_{n}$ and the bias unit *b*:

(11) $$f=\phi \left(\sum_{i=1}^{n}w_{i}x_{i}+bx_{0}\right)=\phi \left(w^{T}x+b\right).$$

**Figure 3:**
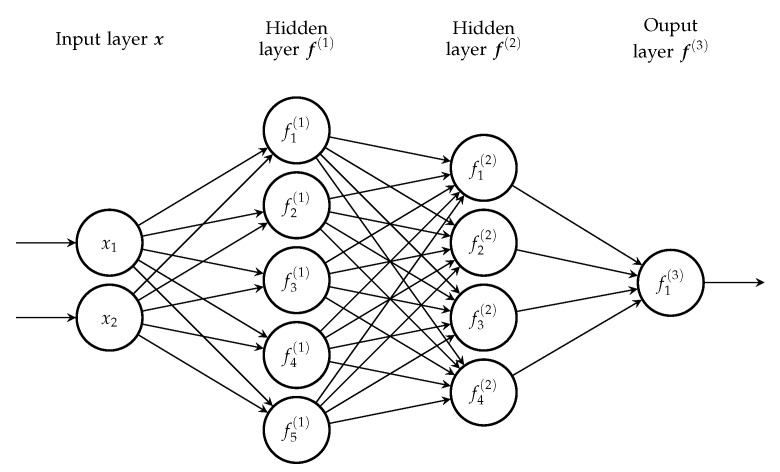
An example of a deep feedforward network; the input layer consists of two neurons, followed by two hidden layers with five and four neurons, respectively, and a single neuron output layer (note: neurons’ bias units are omitted).

Every neuron computes its output *f* using all outputs from the previous layer; such networks are also known as fully connected networks. An example of a feedforward network is shown in Figure 3.

A neural network is essentially a composition of all its layers. The matrix of weights in a layer can be defined as:

(12) $$W={\left[\begin{array}{cccc} w_{1} & w_{2} & \ldots & w_{m} \end{array}\right]}^{T},$$

and the vector of biases as:

(13) $$b={\left[\begin{array}{cccc} b_{1} & b_{2} & \ldots & b_{m} \end{array}\right]}^{T}.$$

Now, the output of an entire layer equals:

(14) $$\begin{array}{cc} f\left(x;W,b\right) & ={\left[\begin{array}{ccc} \phi \left(w_{1}^{T}x+b_{1}\right) & \ldots & \phi \left(w_{m}^{T}x+b_{m}\right) \end{array}\right]}^{T} \end{array}$$

(15) $$\begin{array}{cc} \; & =\phi \left(Wx+b\right). \end{array}$$

Network output is a composition of all layers $f^{\left(i\right)}\left(x;W^{\left(i\right)},b^{\left(i\right)}\right)$, i=1,2,…,L; it follows that the output $\mathrm{dnn}:R^{n}\to R^{m}$ is:

(16) $$\begin{array}{cc} \mathrm{dnn}\left(x;W,B\right) & =\left(f^{\left(1\right)}\circ f^{\left(2\right)}\circ \ldots \circ f^{\left(L\right)}\right) \end{array}$$

(17) $$\begin{array}{cc} \; & =f^{\left(L\right)}\left(f^{\left(L-1\right)}\left(\ldots \left(f^{\left(1\right)}\left(x\right)\right)\ldots \right)\right), \end{array}$$

where $W=\left\{W^{\left(1\right)},W^{\left(2\right)},\ldots ,W^{\left(L\right)}\right\}$ is a set of all weight matrices and $B=\left\{b^{\left(1\right)},b^{\left(2\right)},\ldots ,b^{\left(L\right)}\right\}$ is a set of all bias vectors [36].

Neural networks typically have a very large number of parameters. Their optimal values are obtained through supervised learning by using the backpropagation algorithm, which propagates the error backwards through the network—from the output towards the input layer. The feedforward network provides much flexibility in terms of network configuration, i.e., the number of layers and the number of neurons in each layer. Neural networks are prone to overfitting the training data due to very high model capacity, so good generalization properties are often achieved using some regularization method.

The activation function is another hyperparameter; the most common ones are the sigmoid function, the hyperbolic tangent, the rectifier, etc. It is necessarily non-linear; a network using linear activation functions would be equivalent to a single layer model. The output layer and its activation function are usually defined by the learning task; the sigmoid is well-suited for binary classification problems and yields a probabilistic output that can be used as a PD estimate.

## 4. Performance Measures

**Figure 4:**
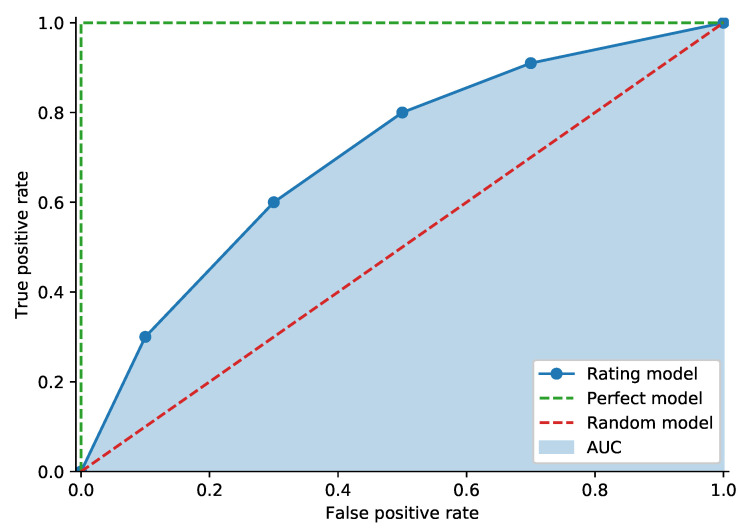
Receiver Operating Characteristic (ROC) curve example.

The Receiver Operating Characteristic curve (ROC curve) is widely used in banking practice. In credit risk terminology, the ROC connects points $\left(x_{r},y_{r}\right)$ for each rating *r* (or classification threshold), where $y_{r}$ is defined as the proportion of bad debtors with a rating worse than or equal to *r*, also called the hit rate or true positive rate. Value $x_{r}$ denotes the proportion of good clients with a rating worse than or equal to *r* (called the false alarm rate or false positive rate). Figure 4 shows an example of an ROC curve. The random model curve sits on the diagonal, and the perfect model connects the origin, (0,1) and (1,1). The perfect model assigns the lowest rating to all bad debtors and higher ratings to all good debtors [1]. It is possible to measure model performance using the Area Under the receiver operating characteristic Curve (AUC). We can obtain the exact value of the AUC by comparing the model outputs of all pairs of defaulted clients $p(x_{D})$ and non-defaulted clients $p(x_{N})$; the score for the individual pair is defined by:

(18) $$S\left(x_{D},x_{N}\right)=\left\{\begin{array}{cc} 1, & \;\mathrm{if}\;p\left(x_{D}\right)>p\left(x_{N}\right)\\ 0.5, & \;\mathrm{if}\;p\left(x_{D}\right)=p\left(x_{N}\right)\\ 0, & \;\mathrm{if}\;p\left(x_{D}\right)<p\left(x_{N}\right) \end{array}\right..$$

The AUC can now be expressed as:

(19) $$\mathrm{AUC}=\frac{1}{N_{D}\cdot N_{N}}\sum_{i=1}^{N_{D}}\sum_{j=1}^{N_{N}}S\left(p\left(x_{D}^{\left(i\right)}\right),p\left(x_{N}^{\left(j\right)}\right)\right),$$

where $N_{D}$ and $N_{N}$ denote the total number of defaulted and non-defaulted examples, respectively. It can be shown that (19) is closely related to the Mann–Whitney–Wilcoxon U statistic [37]. The AUC will be equal to 0.5 in the case of a random model, while the perfect model will have a score of 1.0.

An alternative to the AUC is the H-measure. Reference [38] showed that the AUC is not coherent in terms of misclassification costs, as it uses different misclassification distributions for different classifiers. This implies that the AUC uses a separate metric for each classification model. The proposed H-measure corrects this issue by defining a performance metric that keeps the same cost of misclassification for all classifiers.

We use the Brier score [39] for measuring the accuracy of probability predictions. In the case of binary classification tasks, it has the following formulation:

(20) $$BS=\frac{1}{N}\sum_{i=1}^{N}{\left(y^{\left(i\right)}-p\left(x^{\left(i\right)}\right)\right)}^{2},$$

where $y^{\left(i\right)}$ is the label of the *i*-th example, $p(x^{\left(i\right)})$ denotes the probability of the *i*-th example classified into the positive class, and *N* is the total number of examples. As it is essentially the mean squared error, it follows that the better the predictions are calibrated, the lower the value of the Brier score is.

## 5. Results

As mentioned in Section 2, the last year of both datasets (2009–2013 and 2014–2018) was used for measuring model performance (out-of-time dataset), while the remaining data (in-time sample) were used for model training. Training and hyperparameter optimization were conducted using four-fold cross-validation on the in-time data; the best hyperparameters for each model were chosen based on the highest average AUC score of all four validation splits.

### 5.1. 2009–2013 Dataset

Results for the first dataset with reprograms labeled as defaults are shown in Table 1. Linear models (logistic regression and SVM) exhibited a negligible difference in performance, with logistic regression slightly outperforming linear SVM. As expected, all non-linear models achieved better results than linear ones. The Mean validation AUC was significantly higher, with an at least one percentage point higher out-of-time AUC and up to a five point higher H-measure score. The random forest model had the worst performance among the non-linear models, although having the best calibrated predictions, as indicated by the lowest Brier score (narrowly outperforming XGB). XGB was very evenly matched with the deep feedforward network, with the latter leading in the out-of-time ROC AUC score and the former achieving a higher H-measure and mean validation set ROC AUC. The most significant difference between the two models was their Brier scores, with XGB scoring a much lower 0.039 as opposed to 0.116 for the feedforward model.

Labeling reprograms as non-defaults changed the results significantly; see Table 2. The mean value of the AUC on the validation data generally increased, especially for the two linear models, which experienced a two percentage point jump. The validation performance of non-linear models increased by a more modest 0.5 to 1.5 points. The out-of-time set results demonstrated far greater performance gains. The out-of-time AUC saw a dramatic increase of approximately four percentage points across the board. The H-measure for all models increased by 12–13 points for all models, drastically surpassing the scores in Table 1. Even the Brier scores improved for all models, with random forest still holding the lowest value. The relative performance of the models basically remained unchanged, with XGBoost and the deep model swapping the leading positions for the AUC and H-measure.

### 5.2. 2014–2018 Dataset

Table 3 contains the results for the second dataset with reprograms belonging to the positive class. Once again, we can observe similar performance between linear models, although in this instance, SVM has a slight advantage over the logistic regression. Results for non-linear models are also close, but again, the difference in performance when compared to linear models is obvious with at least 1.5 percentage points for the AUC and more than five points in the case of the H-measure. XGB outperformed other models in both the AUC and H-measure, as well as the mean validation AUC. Next is the deep feedforward model, trailing XGB by a point in the H-measure and less than half percent in the AUC. Random forest had the weakest performance among non-linear models, but once again achieved the lowest Brier score, albeit closely followed by XGB.

Relabeling reprograms as non-defaults did not have a significant impact in the case of the 2014–2018 sample. As shown in Table 4, there is barely any difference between the two labeling methods; logistic regression marked the largest AUC and H-measure gains with a 0.55 and 2.2 percentage point increase, respectively. The overall similarity of the results was expected due to the very low proportion of reprogrammed loans in all defaults (only 1.11% in 2018; Figure 2b). The most notable difference in Table 3 and Table 4 is the mean validation set AUC, which was caused by a significant number of reprogrammed facilities between 2014 and 2016 (59.92% and 64.92% of all defaults, respectively; 2017 data have only 3.89%).

### 5.3. Long-Term Performance

From the perspective of the 2009–2013 development sample, we have five years of additional, unused data that we can use for testing the long-term performance. The results with reprogrammed facilities labeled as defaults are shown in Table 5. The results are fairly similar to the 2013 sample during the first three years with the difference in the AUC below one percentage point in almost all cases. In 2017 and 2018, we see a large jump in performance, once again caused by a small number of reprogrammed examples. AUC values are generally higher, with differences ranging between 3.5 and 4.1 percentage points.

Relabeling reprograms as negative examples yielded much more stable performance during the long-term period; see Table 6. The difference between the maximal and minimal AUC measured for the same model was below 1.5 percentage points in all cases. When compared to the 2013 sample results shown in Table 2, the overall results were remarkably similar to the out-of-time AUC score, with most results within a single percentage point.

Finally, the comparison of Table 5 and Table 6 once again demonstrates a larger difference in the AUC in case of higher proportion of reprogrammed facilities. For 2014, 2015, and 2016, the difference in the AUC varies between 3.2 and 4.1 percentage points, while for the 2017 and 2018, the largest delta barely exceeds one point.

### 5.4. Impact of Reprogrammed Facilities

While it is apparent from performance testing that reprogrammed facilities might be significantly harder to predict due to their noisier nature, we wanted to show that formally. To that end, we decided to test the difference in the mean probability of default assigned to reprogrammed facilities and the mean PD of defaulted loans. We used 2013 data for the test, as that sample had a significant proportion of reprograms in all defaults (50.78%). As for the PD estimates, we decided to test both the deep feedforward model and XGB, as they were the top two models in terms of general performance.

We used a one-tailed *t*-test with hypothesis $H_{0}:{\bar{\mathrm{PD}}}_{\mathrm{def}}\leq {\bar{\mathrm{PD}}}_{\mathrm{rep}}$. The sample sizes were not equal (3552 defaults and 3664 reprograms), and we did not assume equal variances. For both the deep model and XGB, the results of the test show that the null hypothesis can be rejected at the level of significance α=0.01 in favor of the alternative ${\bar{\mathrm{PD}}}_{\mathrm{def}}>{\bar{\mathrm{PD}}}_{\mathrm{rep}}$. This confirms the statistical significance of the assumption that reprogrammed facilities are, on average, harder to predict than regular defaults, with a greater average PD of defaulted loans when compared to the average PD of reprograms.

**Figure 5:**
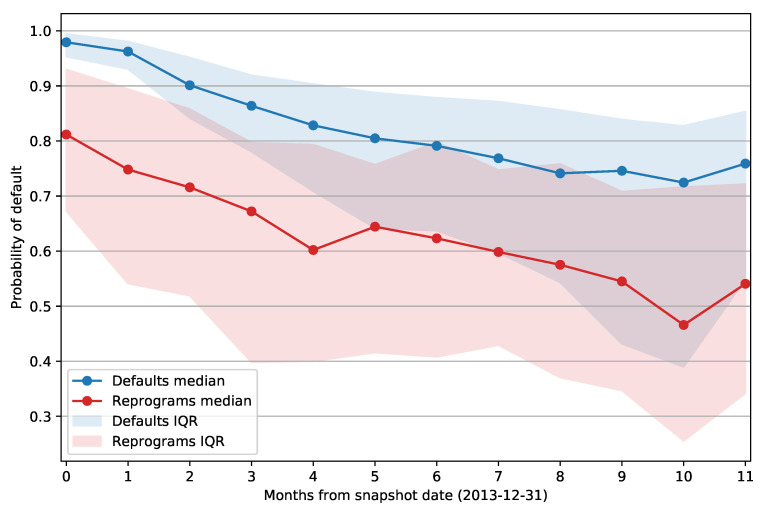
Deep neural network model: median Probability of Default (PD) of defaulted and reprogrammed loans based on the number of months from the snapshot date to the opening of the default status or reprogram. zero months represents a period between one and 30 days; one month is 31 to 60 days, etc. The width of envelopes around the curves represents the Interquartile Range (IRQ) for each data point.

Figure 5 shows the median PD of defaulted and reprogrammed facilities grouped by number of months passed between the snapshot date and the opening of the default status or reprogram. The envelope around the curve represents the interquartile range for each data point. It is apparent that the same difference between the mean PD values holds if we split the facilities based on the days past from the snapshot date; reprogrammed examples again have a lower PD when compared to defaults. Note also that the interquartile range of reprogram PDs does not change over time as significantly as the IQR of defaulted facilities. The PD of defaults has an especially high median value and low IQR for facilities that defaulted within 60 days from the snapshot date (first two data points). This is expected as those clients are at least 30 days past due on their obligations, and that information will be present in the features that describe the client’s credit history. As the month delta increases, we can observe a downward trend in the median PD of both defaulted and reprogrammed facilities, and default PDs exhibit a significant increase in IQR as the model outputs become less accurate.

### 5.5. Distribution of PD Estimates

**Figure 6:**
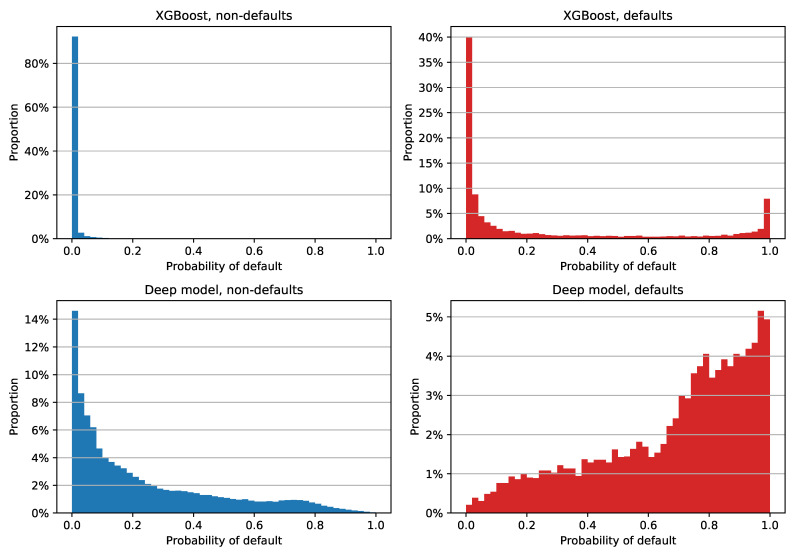
Distribution of out-of-time PD estimates for XGBoost and the deep model on 2013-12-31 data; both models were trained on 2009–2012 data with reprograms labeled as defaults. The histogram for non-defaulted examples is shown in the left column, while defaulted examples are in the right one. The top row contains XGBoost PDs, while the deep model estimates are in the bottom row. The width of each histogram column is two percentage points.

In order to check PDs for different labeling of reprograms and to gain more insight into the difference in Brier score between the XGBoost and deep models, we decided to examine the histogram of PD estimates for each class and each model separately. The plot for reprograms labeled as defaults is shown in Figure 6. We can see that the XGBoost model assigned a low PD to a large majority of non-defaulted examples: more than 80% of them have a PD between 0% and 2% (Figure 6, upper left). Although the estimates of non-defaulted examples look very good, the positive class PDs are not as impressive; see Figure 6, upper right. Most of the defaulted facilities were assigned a PD close to zero, which is obviously not the desired model behavior for this class. This tendency of assigning low PDs regardless of the class is the main reason why XGBoost has a low Brier score: the out-of-time sample has a default rate of 4.91%, which means that the PD estimate errors of the defaulted examples do not have a significant impact on the overall Brier score. XGBoost’s median PD on the entire out-of-time sample is 0.062%, while non-defaulted and defaulted examples have median PDs of 0.054% and 4.435%, respectively.

When compared to XGBoost estimates, the deep model PDs are not as tightly grouped; see Figure 6, bottom row. We can see that the model has a clear tendency of assigning greater PDs to defaults and lower estimates to negative examples. This is reflected in the median PD values as well, with a 16.067% overall median estimate, 14.645% for the non-defaulted, and 75.597% for the defaulted class.

A simple way to quantify the difference in the accuracy of probability predictions for each class is to compute the Brier score for each class separately. The results are shown in Table 7. We can see that XGBoost has a lower Brier score on the entire dataset and on non-defaulted examples. However, in the case of defaulted examples, XGBoost has a very high Brier score of 0.6757. The deep feedforward model exhibited much more stable values, with a score of 0.1642 for defaults only. If we simply averaged the Brier scores for individual classes, which would effectively give the same importance to each class (instead of each example), we would get a completely different result: XGBoost would score 0.3410, and the deep feedforward model would have a much lower value of 0.1390.

**Figure 7:**
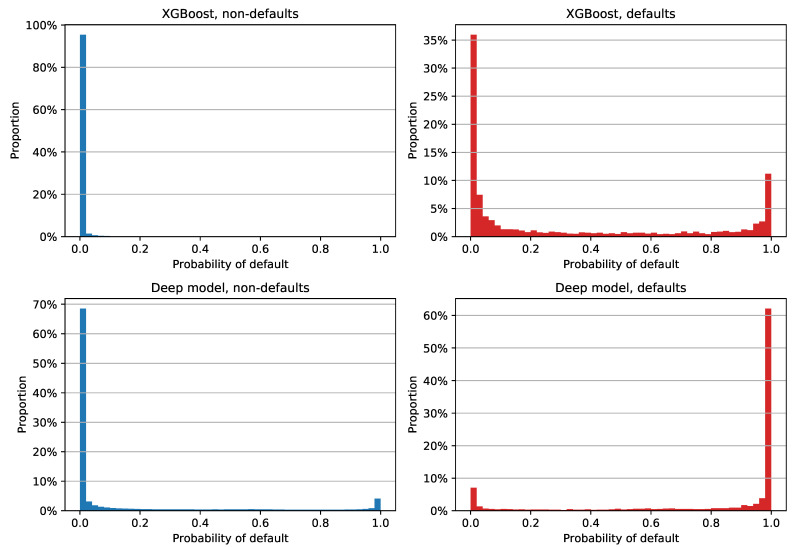
Distribution of out-of-time PD estimates for XGBoost and the deep model on 2013-12-31 data; both models were trained on 2009–2012 data with reprograms labeled as non-defaults. The histogram for non-defaulted examples is shown in the left column, while defaulted examples are in the right one. The top row contains XGBoost PDs, while the deep model estimates are in the bottom row. The width of each histogram column is two percentage points.

Results with reprograms labeled as non-defaults are shown in Figure 7. XGBoost estimates are fairly similar to the previous plot, with somewhat different median PD values: 0.020%, 0.019%, and 8.150% for all examples, non-defaults, and defaults, respectively. The deep model’s estimates are clearly different: more than 60% of non-defaults were assigned a PD between 0% and 2%, while more than 60% of defaults had an estimate between 98% and 100%. In terms of the median PDs, the neural network had an overall value of 0.031%, with non-defaults’ and defaults’ median estimates of 0.024% and 99.844%, respectively. Still, it is apparent from Figure 7 that the deep model had 4.08% of non-defaulted examples that had a very large PD and 7.04% of defaults that were assigned very small PD estimates. The former error had an especially negative impact on the Brier score, since labeling reprograms as non-defaults lowered the default rate to 2.48%.

The Brier scores for individual classes with reprograms labeled as non-defaults are shown in Table 8. The results are fairly similar to the previous scenario; XGBoost achieved an overall lower score thanks to the combination of low PDs regardless of the class and 97.52% negative examples, while the positive class once again scored a very high 0.6010. The deep model achieved lower variance between scores, with a positive class Brier score of 0.1424. If we averaged the values for individual classes, the neural network had a mean score of 0.1249, which is a much better result than 0.3029 in the case of XGBoost.

### 5.6. Results Summary

In this paper, we developed a deep learning model for behavioral credit risk assessment. In order to measure model performance in different scenarios, we split the available data into two parts: during the financial crisis (2009–2013) and post financial crisis (2014–2018). The last year of both datasets was left out for testing purposes on examples that were time-disjoint from the model development sample. Additionally, we wanted to demonstrate the impact of reprogrammed loans that are in general considered to be noisy and harder to predict than regular defaults. To that end, each of the datasets had a version where reprograms were labeled as defaults and another where reprograms were in the negative class.

As proposed by [18], we used several measures for model evaluation: ROC AUC, H-measure, and Brier score. We used multiple benchmark models, logistic regression and SVM as linear, and two non-linear tree based models: random forest and XGBoost. All models were trained on both 2009–2013 and 2014–2018 datasets.

Unsurprisingly, the non-linear models outperformed their linear counterparts regardless of the dataset. Comparing linear models only, logistic regression and SVM had remarkably similar performance. As for non-linear models, XGB and the feedforward network were evenly matched in all scenarios, with XGB having a slight edge on the deep model in most cases. Random forest was placed third, with overall middle of the pack performance in all categories except for the consistently lowest Brier scores.

We also showed that the classification of reprogrammed loans poses a greater challenge than defaulted facilities. This is an expected result considering that they can be quite unpredictable, as clients’ financial circumstances can take an abrupt turn for the worse in a short amount of time before they apply for a reprogrammed loan; that kind of change might not be present in the data. Labeling reprogrammed examples as non-defaults yielded significantly more stable results, with long-term ROC AUC scores within a percentage point regardless of the model and varying default rate.

It is apparent that there is a significant performance gain in using non-linear models as opposed to linear ones such as logistic regression or SVM. The developed deep neural network outperformed all benchmarks except XGBoost. As those two models demonstrated evenly matched performance, we would recommend using either of them as a benchmark for future, more advanced model development. Although the random forest model was recommended in [18], in our testing, it did not perform on par with the DNN and XGB.

As demonstrated in Section 5.5, a low Brier score does not necessarily imply accurate probability predictions for individual classes. XGBoost had a better overall Brier score when compared to the deep model, but if we measured positive class prediction error, the neural network achieved significantly better scores. Whether this is an issue or not depends on the use case and the researchers’ preferences.

## 6. Conclusions

The presented deep neural network model for behavioral credit risk assessment provided the expected performance gain when compared to linear benchmark models. Moreover, all three non-linear models that we trained managed to deliver better performance than their linear counterparts, with either the deep model or XGBoost achieving the best results.

In our view, there is no clear winner between the two as it is not uncommon that one model had a better AUC score, while the other had a higher H-measure. The Brier score complicates things even further: although its value suggests a more precise probability estimate on the whole dataset, it can be poorly calibrated to the minority class. Based on the presented results, it is our recommendation that researchers and practitioners should decide which performance measures are the most important ones for their use case and choose the better model accordingly.

We believe that the demonstrated difference in performance is significant enough to be beneficial to both banks and clients alike, so it would make long-term sense to reconsider the regulatory requirements for model explainability and to allow the usage of non-linear models for credit risk assessment purposes.

Finally, we would recommend taking reprogrammed examples into account separately. Labeling them as both positive and negative examples should give researchers insight into their impact on the stability of model performance, as treating them as defaults managed to lower the ROC AUC score over a long period of time and for all models.

## Figures and Tables

**Table 1:** Performance of the models trained on 2009–2012 data; reprogrammed loans are labeled as defaults.

Model	Validation Set	Out-of-Time Set (2013-12-31)		
	Mean ROC AUC	ROC AUC	H-Measure	Brier Score
Logistic regression	0.896668	0.866566	0.414292	0.124405
Linear SVM	0.896090	0.865722	0.413397	-
Random forest	0.939872	0.878587	0.441497	**0.037041**
XGBoost	**0.940979**	0.886009	**0.456540**	0.039166
Deep feedforward	0.914695	**0.886477**	0.456309	0.116189

**Table 2:** Performance of the models trained on 2009–2012 data; reprogrammed loans are labeled as non-defaults.

Model	Validation Set	Out-of-Time Set (2013-12-31)		
	Mean ROC AUC	ROC AUC	H-Measure	Brier Score
Logistic regression	0.916323	0.906946	0.543300	0.091685
Linear SVM	0.916179	0.908359	0.548104	-
Random forest	0.944355	0.917116	0.564419	**0.018484**
XGBoost	**0.948748**	**0.921723**	0.573775	0.019638
Deep feedforward	0.928784	0.920317	**0.578402**	0.108295

**Table 3:** Performance of the models trained on 2014–2017 data; reprogrammed loans are labeled as defaults.

Model	Validation Set	Out-of-Time Set (2018-12-31)		
	Mean ROC AUC	ROC AUC	H-Measure	Brier Score
Logistic regression	0.896018	0.909755	0.547925	0.073841
Linear SVM	0.895249	0.910149	0.553930	-
Random forest	0.951180	0.925821	0.604190	**0.013256**
XGBoost	**0.953976**	**0.933554**	**0.618382**	0.013580
Deep feedforward	0.917070	0.929786	0.612123	0.054511

**Table 4:** Performance of the models trained on 2014–2017 data; reprogrammed loans are labeled as non-defaults.

Model	Validation Set	Out-of-Time Set (2018-12-31)		
	Mean ROC AUC	ROC AUC	H-Measure	Brier Score
Logistic regression	0.922209	0.915283	0.570536	0.079378
Linear SVM	0.921126	0.914567	0.569720	-
Random forest	0.958128	0.925179	0.602819	**0.013188**
XGBoost	**0.961277**	**0.933961**	**0.618473**	0.013683
Deep feedforward	0.939366	0.933304	0.615086	0.084993

**Table 5:** Long-term ROC AUC score of the models trained on 2009–2012 data with reprogrammed loans labeled as defaults.

Model	Out-of-Time Set				
	2014-12-31	2015-12-31	2016-12-31	2017-12-31	2018-12-31
Logistic regression	0.873557	0.874864	0.869676	0.906084	0.905157
Linear SVM	0.874060	0.875146	0.871298	0.906778	0.906335
Random forest	0.886108	0.889226	0.878379	0.919961	0.917771
XGBoost	0.892793	**0.896854**	**0.888610**	**0.926328**	**0.925317**
Deep feedforward	**0.893153**	0.895266	0.885186	0.925506	0.921958

**Table 6:** Long-term ROC AUC score of the models trained on 2009–2012 data with reprogrammed loans labeled as non-defaults.

Model	Out-of-Time Set				
	2014-12-31	2015-12-31	2016-12-31	2017-12-31	2018-12-31
Logistic regression	0.914545	0.912980	0.907418	0.914217	0.906431
Linear SVM	0.915236	0.915165	0.908992	0.917692	0.909679
Random forest	0.925358	0.927714	0.912753	0.927178	0.922232
XGBoost	**0.931065**	**0.932262**	**0.921962**	**0.932984**	**0.931460**
Deep feedforward	0.928203	0.927673	0.920908	0.931293	0.924273

**Table 7:** Out-of-time (2013-12-31) Brier scores for all examples and individual classes; models were trained on 2009–2012 data, with reprograms labeled as defaults.

Model	Brier Score		
	All Examples	Non-Defaults	Defaults
XGBoost	**0.039166**	**0.006279**	0.675704
Deep feedforward	0.116189	0.113707	**0.164224**

**Table 8:** Out-of-time (2013-12-31) Brier scores for all examples and individual classes; models were trained on 2009–2012 data, with reprograms labeled as non-defaults.

Model	Brier Score		
	All Examples	Non-Defaults	Defaults
XGBoost	**0.019638**	**0.004879**	0.600952
Deep feedforward	0.108295	0.107429	**0.142368**
