# Addendum: Merćep, A., et al. Deep Neural Networks for Behavioral Credit Rating. *Entropy* 2021, *23*, 27

**DOI:** 10.3390/e23030330

**Published:** 2021-03-11

**Authors:** Andro Merćep, Lovre Mrčela, Matija Birov, Zvonko Kostanjčar

**Affiliations:** 1 Laboratory for Financial and Risk Analytics, Faculty of Electrical Engineering and Computing, University of Zagreb, 10000 Zagreb, Croatia; lovre.mrcela@fer.hr (L.M.); zvonko.kostanjcar@fer.hr (Z.K.); 2 Privredna Banka Zagreb, Member of Intesa Sanpaolo Group, 10000 Zagreb, Croatia; matija.birov@pbz.hr

The authors would like to add the following information to the “Funding” section of their paper [1]:

“This work was supported in part by the Croatian Science Foundation under Project 5241 and in part by the European Regional Development Fund under Grant KK.01.1.1.01.0009 (DATACROSS).”

The authors would also like to apologize to the readers for any inconvenience caused by this change.
